# Circulating adiponectin levels in various malignancies: an updated meta-analysis of 107 studies

**DOI:** 10.18632/oncotarget.8932

**Published:** 2016-04-22

**Authors:** Tai Wei, Peng Ye, Xin Peng, Li-Ling Wu, Guang-Yan Yu

**Affiliations:** ^1^ Department of Oral and Maxillofacial Surgery, Peking University School and Hospital of Stomatology, Beijing, China; ^2^ Department of Physiology and Pathophysiology, Peking University Health Science Center, Key Laboratory of Molecular Cardiovascular Sciences, Ministry of Education, and Beijing Key Laboratory of Cardiovascular Receptors Research, Beijing, China

**Keywords:** adiponectin, malignancy, biomarker, diagnosis, meta-analysis

## Abstract

Early detection of cancers is challenging for lack of specific biomarkers. Adiponectin is an adipokine predominantly derived from adipocytes and hypoadiponectinemia has been reported to associate with risk of many types of cancers. However, available evidence is controversial. Some studies show that increased adiponectin levels correlate with cancer risk. Therefore, we performed a meta-analysis of the association between circulating adiponectin levels and cancer development. A systematic search of PubMed, EMBASE, Wiley Online Library and Cochrane Library was conducted for eligible studies involving circulating adiponectin and malignancies from inception to August 8, 2015. Standard mean differences (SMDs) with 95% confidence intervals (95% CIs) were calculated by use of a random-effect model. Funnel plot and Egger's linear regression test were conducted to examine the risk of publication bias. 107 studies were included with 19,319 cases and 25,675 controls. The pooled analysis indicated that circulating adiponectin levels were lower in patients with various cancers than in controls, with a pooled SMD of −0.334 μg/ml (95% CI, −0.465 to −0.203, *P* = 0.000). No evidence of publication bias was observed. Circulating high molecular weight adiponectin levels were also lower in cancer patients than in controls, with a pooled SMD of −0.502 μg/ml (95% CI, −0.957 to −0.047, *P* = 0.000). This meta-analysis provides further evidence that decreased adiponectin levels is associated with risk of various cancers. Hypoadiponectinemia may represent a useful biomarker for early detection of cancers.

## INTRODUCTION

Cancer, a major cause of human mortality, has been a worldwide public health problem. A variety of factors such as genetic lesions, environmental aspect and increasing adoption of unhealthy lifestyle are considered as crucial causes of cancer [1]. Among them, obesity is an important factor contributing to the occurrence and development of malignancies. According to the literature in 2005, 396 and 937 million people suffer from obese and overweight worldwide, respectively [2]. Epidemiological research reveals that obesity increases the risk of cancer with evidence that obese women have 50% higher incidence rate than normal weight women [3]. In the process of obesity, dysregulated circulating hormones and growth factors may play an important role in carcinogenesis [4]. Among them, aberrant adiponectin concentration is reported to be a vital link between obesity and cancer.

Adiponectin, firstly discovered by Scherer *et al.* in 1995, is an adipokine predominantly produced by adipocytes with the monomeric subunit containing 244 amino-acids in human and circulates abundantly in plasma [5, 6]. Three bioactive forms of adiponectin are produced after post-transcriptional process known as trimeric low molecular weight (90 kD, LMW), hexameric medium molecular weight (180 kD, MMW) and oligomeric high molecular weight (> 400 kD, HMW) adiponectin. Among them, HMW-adiponectin is the dominant form in plasma and has the most biological activity than the other two isoforms [7]. Adiponectin mainly acts on two seven-transmembrane adiponectin receptors, AdipoR1 and AdipoR2. Besides, T-cadherin is also responsible for mediating the role of adiponectin in certain tissues [8, 9]. Adiponectin exerts pleiotropic functions in human health such as anti-inflammation, anti-atherosclerosis, and anti-angiogenesis. It also has the properties of insulin-sensitizing and balancing glucose and lipid metabolism in various cells [10]. A number of studies reveal that circulating adiponectin levels decrease in metabolic syndrome, whereas overexpression of it can counteract metabolic dysfunctions [10]. Besides, increased weight reduces the plasma adiponectin level and decreased weight upregulates circulating adiponectin level [11].

It was first reported that circulating adiponectin level was lower in patients with breast cancer in 2003 [12]. Since then, the most clinical studies have indicated that hypoadiponectinemia is associated with risk of various cancers including prostate, endometrial, and colorectal cancers [13–16]. In addition, adiponectin has anti-proliferative and pro-apoptotic effects on cultured cancer cell lines [17, 18]. These results suggest that adiponectin might be an important regulator in carcinogenesis and progression of cancers. However, unchanged or increased circulating adiponectin levels in pancreatic and hepatocellular carcinoma are also reported [19, 20]. Therefore, understanding the exact role of adiponectin in cancer may offer a novel target in tumor diagnosis and therapeutic strategy. In order to gain a more explicit and evidence-based conclusion on the association between circulating adiponectin levels and carcinogenesis, we conducted a comprehensive meta-analysis of current available studies.

## RESULTS

### Literature selection

**Figure 1:**
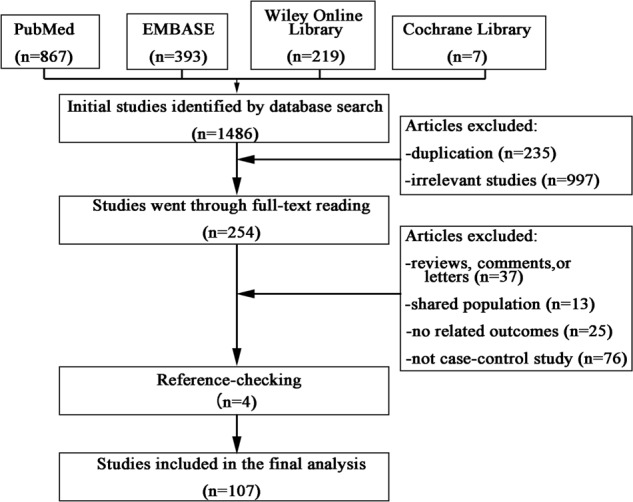
Flow diagram of the included studies

The initial comprehensive search yielded 1486 articles, of which 235 articles were excluded for duplication. Then 997 studies were ruled out because of apparent irrelevance after reading titles and/or abstracts. The remaining 254 studies were included for full-text reading, of which 151 studies were removed for one of the following reasons: (i) reviews, comments or letters (n = 37); (ii) shared population (n = 13); (iii) no report of adiponectin levels and/or SDs for both patients and controls or there was not enough information to calculate them (n = 25); (iv) not case-control study (n = 76). 4 additional studies were included from checking the references list. Finally, 107 studies met the inclusion criteria and were used for further analysis [12, 13, 15, 16, 20–112]. The flow diagram of this selection process was showed in Figure 1.

### Study characteristics

Among the 107 studies, a total of 25,675 controls and 19,319 cases were enrolled until August, 2015. Geographic regions were various, among which 46 studies from Asia, 39 studies from Europe, 19 studies from America, and 3 studies from Africa. 16 types of malignancies were investigated in this meta-analysis, with digestive system cancers accounting for the largest percentage (43 studies); other types included: breast cancer (20 studies), prostate cancer (13 studies), endometrial carcinoma (11 studies), lung cancer (5 studies), renal cancer (3 studies), acute leukemia (3 studies), non-Hodgkin's lymphoma (3 studies), Hodgkin's lymphoma (1 study), multiple myeloma (2 studies), melanoma (1 study), thyroid cancer (1 study), and tongue cancer (1 study). Circulating samples included serum (65 studies) and plasma (37 studies), while 5 studies did not mention the exact one. Most researches provided the mean concentrations of circulating adiponectin levels and the SDs of them. SDs from 11 studies were calculated based on the sample size and *P* values. 96 studies had NOS scores greater than 6 along with 11 studies had scores of 5. The main characteristics of eligible articles were listed in Table 1.

**Table 1:** Characteristics of all the included studies in the meta-analysis

Author	Year	Type	Country	Ethnicity	Sample	Mean age (Case/control)	Number (Case/control)	Study design	Assay method	Assay source	Study quality
Petridou et al.	2006	Acute leukemia	Greece/USA	Caucasian	Serum	NR	201/201	Case-control	RIA	Beth Israsel Deaconess Medical Center	8
Moschovi et al.	2010	Acute leukemia	Greece	Caucasian	Plasma	4.3/5.2	9/9	Prospective case-control	Other	Linco Research	7
Aref et al.	2013	Acute leukemia	Egypt	African	Serum	42.8/49.1	80/20	Case-control	Elisa	R&D Systems	5
Miyoshi et al.	2003	Breast cancer	Japan	Asian	Serum	54.0/52.8	102/100	Case-control	Elisa	NR	7
Mantzoros et al.	2004	Breast cancer	Greece	Caucasian	Serum	NR	174/167	Case-control	RIA	Beth Israsel Deaconess Medical Center	8
Chen et al.	2006	Breast cancer	Taiwan	Asian	Serum	49.9/48.9	100/100	Case-control	RIA	Linco Research	7
Korner et al.	2007	Breast cancer	Greece	Caucasian	Serum	62.5/55.6	74/76	Case-control	RIA	ALPCO Diagnostics	7
Kang et al.	2007	Breast cancer	Korea	Asian	Serum	47.4/47.8	41/43	Case-control	Elisa	AdipoGen	7
Hou et al.	2007	Breast cancer	China	Asian	Serum	48/49	80/50	Case-control	Elisa	R&D Systems	6
Tworoger et al.	2007	Breast cancer	USA	Caucasian	Blood	57.1/58.1	1166/1575	Nested case-control	RIA	Linco Research	7
Tworoger et al.	2007	Breast cancer	USA	Caucasian	Blood	45.4/45.1	311/621	Nested case-control	RIA	Linco Research	7
Hancke et al.	2010	Breast cancer	Switzerland	Caucasian	Serum	59.5/49.0	159/41	Case-control	Elisa	BioVendor Laboratory Medicine	6
Cust et al.	2009	Breast cancer	Sweden	Caucasian	Plasma	52.5/NR	561/561	Case-control	RIA	Linco Research	7
Shahar et al.	2010	Breast cancer	Malaysia	Asian	Serum	47.3/46.2	70/138	Case-control	Elisa	Linco Research	7
Dalamaga et al.	2011	Breast cancer	Greece	Caucasian	Serum	61.5/62.8	102/102	Case-control	Elisa	Avibion	7
Al Khaldi et al.	2011	Breast cancer	Kuwait	Asian	Plasma	49/60	60/68	Case-control	Elisa	Linco Research	7
Touvier et al.	2013	Breast cancer	France	Caucasian	Plasma	49.2/51.5	218/436	Nested case-control	Elisa	R&D Systems	9
Gulcelik et al.	2012	Breast cancer	Turkey	Asian	Serum	51.4/52.4	83/40	Case-control	Elisa	B-Bridge International Inc.	7
Al Awadhi et al.	2012	Breast cancer	Kuwait	Asian	Plasma	50.3/50.7	144/77	Case-control	Elisa	Linco Research	7
Alokail et al.	2013	Breast cancer	Saudi Arabia	Asian	Serum	46.4/43.1	56/53	Case-control	Other	Luminex Corporation	7
Ollberding et al.	2013	Breast cancer	USA	Caucasian	Serum	67.8/67.8	706/706	Nested case-control	Elisa	R&D Systems	8
Gross et al.	2013	Breast cancer	USA	Caucasian	Plasma	62.6/62.5	272/272	Case-control	Elisa	ALPCO Diagnostics	7
Minatoya et al.	2014	Breast cancer	Japan	Asian	Serum	NR	66/66	Case-control	Other	SRL	7
Gulcelik et al.	2012	Colon cancer	Turkey	Asian	Serum	52.1/52.4	27/40	Case-control	Elisa	B-Bridge International Inc.	7
Otake et al.	2005	Colorectal adenoma	Japan	Asian	Plasma	59.0/58.0	51/52	Case-control	Elisa	Otsuka Pharmaceutical	8
Fukumoto et al.	2008	Colorectal adenoma	Japan	Asian	Plasma	NR	656/648	Case-control	Elisa	Otsuka Pharmaceutical	7
Kumor et al.	2009	Colorectal adenoma	Poland	Caucasian	Serum	62.4/60.1	37/25	Case-control	Elisa	R&D Systems	7
Erarslan et al.	2009	Colorectal adenoma	Turkey	Asian	Plasma	63.0/59.0	31/50	Case-control	Elisa	RayBio	8
Nakajima et al.	2010	Colorectal adenoma	Japan	Asian	Plasma	66.8/66.7	72/72	Case-control	Elisa	Otsuka Pharmaceutical	7
Otake et al.	2010	Colorectal adenoma	Japan	Asian	Plasma	65.1/67.9	47/26	Case-control	Elisa	Otsuka Pharmaceutical	7
Yamaji et al.	2010	Colorectal adenoma	Japan	Asian	Plasma	NR	778/735	Case-control	Elisa	Sekisui Medical	6
Danese et al.	2013	Colorectal adenoma	Italy	Caucasian	Serum	63.0/59.5	40/40	Case-control	Elisa	Mediagnost	7
Wei et al.	2005	Colorectal cancer	USA	Caucasian	Plasma	66.6/66.5	179/356	Nested case-control	RIA	Linco Research	8
Stocks et al.	2008	Colorectal cancer	Sweden	Caucasian	Plasma	59.7/NR	306/595	Nested case-control	Elisa	R&D Systems	6
Guadagni et al.	2009	Colorectal cancer	Italy	Caucasian	Serum	63.0/59.0	90/30	Case-control	Elisa	BioVendor Laboratory Medicine	8
Kumor et al.	2009	Colorectal cancer	Poland	Caucasian	Serum	58.6/60.1	36/25	Case-control	Elisa	R&D Systems	7
Erarslan et al.	2009	Colorectal cancer	Turkey	Asian	Plasma	57.0/59.0	23/50	Case-control	Elisa	RayBio	8
Nakajima et al.	2010	Colorectal cancer	Japan	Asian	Plasma	63.7/63.5	115/115	Case-control	Elisa	Otsuka Pharmaceutical	7
Otake et al.	2010	Colorectal cancer	Japan	Asian	Plasma	66.7/67.9	51/26	Case-control	Elisa	Otsuka Pharmaceutical	7
Kemik et al	2010	Colorectal cancer	Turkey	Asian	Serum	43.5/40.4	126/38	Case-control	RIA	Linco Research	7
Gonullu et al.	2010	Colorectal cancer	Turkey	Asian	Serum	56.6/51.0	36/37	Case-control	Elisa	BioSource	8
Catalan et al.	2011	Colorectal cancer	Spain	Caucasian	Plasma	66.0/44.0	11/18	Case-control	Elisa	R&D Systems	8
Chen et al.	2012	Colorectal cancer	China	Asian	Plasma	61.9/58.3	165/102	Case-control	Elisa	Adlitteram Diagnostic Laboratories. Inc.	7
Touvier et al.	2012	Colorectal cancer	France	Caucasian	Plasma	51.8/52.1	50/100	Nested case-control	Elisa	R&D Systems	9
Aleksandrova et al.	2012	Colorectal cancer	Germany	Caucasian	Serum	58.3/58.3	1206/1206	Case-control	Elisa	ALPCO Diagnostics	9
Song et al.	2013	Colorectal cancer	USA	Caucasian	Plasma	61.9/61.9	616/1205	Case-control	Elisa	ALPCO Diagnostics	9
Cust et al.	2007	Endometrical carcinoma	UK	Caucasian	Plasma	56.9/56.9	284/548	Nested case-control	Elisa	R&D Systems	8
Soliman et al.	2006	Endometrical carcinoma	USA	Caucasian	Serum	66.6/61.2	117/238	Case-control	Elisa	R&D Systems	5
Ashizawa et al.	2010	Endometrical carcinoma	Japan	Asian	Serum	59.9/57.5	146/150	Case-control	RIA	Linco Research	8
Dossus et al.	2013	Endometrical carcinoma	Germany	Caucasian	Serum	57.7/57.7	233/446	Case-control	Elisa	R&D Systems	8
Friedenreich et al.	2012	Endometrical carcinoma	USA	Caucasian	Serum	59/59	514/962	Case-control	Elisa	ALPCO Diagnostics	9
Luhn et al.	2013	Endometrical carcinoma	USA	Caucasian	Serum	NR	167/327	Nested case-control	RIA	Linco Research	8
Erdogan et al.	2013	Endometrical carcinoma	Turkey	Asian	Serum	56.6/49.7	60/70	Case-control	Elisa	eBioscience	6
Ma et al.	2013	Endometrical carcinoma	China	Asian	Serum	53.2/53.3	206/310	Case-control	Elisa	Bender MedSystems	9
Dallal et al.	2013	Endometrical carcinoma	USA	Caucasian	Serum	67.4/67.5	62/124	Nested case-control	Elisa	Millipore	8
Mihu et al.	2013	Endometrical carcinoma	Romania	Caucasian	Serum	60.2/58.5	44/44	Case-control	Elisa	R&D Systems	6
Ohbuchi et al.	2014	Endometrical carcinoma	Japan	Asian	Serum	61.2/58.1	43/62	Case-control	Elisa	Daiichi Co. Ltd.	8
Diao et al.	2009	Esophageal cancer	China	Asian	Plasma	58.0/49.0	43/33	Case-control	Elisa	Adlitteram Diagnostic Laboratories. Inc.	6
Nakajima et al.	2010	Esophageal cancer	Japan	Asian	Blood	63.6/63.6	117/117	Case-control	Elisa	Otsuka Pharmaceutical	6
Yildirim et al.	2009	Esophageal cancer	Turkey	Asian	Serum	64/61	62/30	Case-control	Elisa	Avibion	6
Ishikawa et al.	2005	Gastric cancer	Japan	Asian	Plasma	64.2/59.3	75/52	Case-control	Elisa	Otsuka Pharmaceutical	6
Nakajima et al.	2009	Gastric cancer	Japan	Asian	Blood	61.0/60.8	156/156	Case-control	Elisa	Otsuka Pharmaceutical	8
Seker et al.	2010	Gastric cancer	Turkey	Asian	Plasma	60.0/38.6	40/43	Case-control	Elisa	Linco Research	5
Diakowska et al.	2014	Gastroesophageal cancer	Poland	Caucasian	Serum	60.0/58.0	85/60	Case-control	Elisa	R&D Systems	7
Kotani et al.	2009	Hepatacellular carcinoma	Japan	Asian	Serum	63.5/62.7	59/334	Nested case-control	Elisa	Daiichi Co. Ltd.	8
Liu et al.	2009	Hepatacellular carcinoma	Taiwan/China	Asian	Serum	50.7/53.8	120/116	Case-control	Elisa	B-Bridge International Inc.	5
Sumie et al.	2011	Hepatacellular carcinoma	Japan	Asian	Serum	67.4/61.2	97/97	Case-control	Elisa	EikenChenical Co. Ltd.	7
Sadik et al	2012	Hepatacellular carcinoma	Egypt	African	Serum	58.9/55.7	69/121	Case-control	Elisa	Assaypro	7
Chen et al.	2012	Hepatacellular carcinoma	Taiwan/China	Asian	Serum	52.4/52.2	65/165	Case-control	RIA	Linco Research	6
Khattab et al.	2012	Hepatacellular carcinoma	Egypt	African	Plasma	43.9/42.9	147/320	Case-control	Other	Linco Research	5
Chen et al.	2014	Hepatacellular carcinoma	Taiwan/China	Asian	Plasma	NR	185/373	Nested case-control	Elisa	B-Bridge International Inc.	8
Petridou et al.	2010	Hodgkin lymphoma	Greece	Caucasian	Serum	11.5/11.2	75/75	Case-control	RIA	Linco Research	7
Jamieson et al.	2004	Lung cancer	UK	Caucasian	Serum	64.0/65.0	20/13	Case-control	RIA	Linco Research	7
Karapanagiotou et al.	2008	Lung cancer	Greece	Caucasian	Serum	64.2/55.5	101/51	Case-control	Elisa	BioVendor	6
Petridou et al.	2007	Lung cancer	Greece	Caucasian	Serum	NR	85/170	Case-control	RIA	Beth Israsel Deaconess Medical Center	8
Gulen et al.	2012	Lung cancer	Turkey	Asian	Serum	65.6/63.5	63/25	Case-control	Elisa	BioVendor	7
Kerenidi et al.	2013	Lung cancer	Greece	Caucasian	Serum	62.9/NR	80/40	Case-control	Elisa	Linco Research	7
Antoniadis et al.	2011	Melanoma	Greece/Canada	Caucasian	Serum	52.7/53.3	55/165	Case-control	RIA	Beth Israsel Deaconess Medical Center	8
Dalamaga et al.	2009	Multiple myeloma	Greece/Canada	Caucasian	Serum	NR	73/73	Case-control	Elisa	Avibion	8
Hofmann et al.	2012	Multiple myeloma	USA	Caucasian	Plasma	NR	174/348	Case-control	Elisa	R&D Systems	7
Pamuk et al.	2006	Non-Hodgkin's lymphoma	Turkey	Asian	Serum	63.2/58.5	28/17	Case-control	Elisa	OtsukaCo.Ltd	5
Petridou et al.	2009	Non-Hodgkin's lymphoma	Greece	Caucasian	Serum	8.8/8.8	121/121	Case-control	RIA	NR	7
Conroy et al.	2013	Non-Hodgkin's lymphoma	USA	Caucasian	Plasma	70.0/70.0	272/541	Nested case-control	Elisa	R&D Systems	7
Chang et al.	2007	Pancreatic cancer	Taiwan/China	Asian	Serum	64.6/49.5	72/290	Case-control	Elisa	R&D Systems	8
Dalamaga et al.	2009	Pancreatic cancer	Greece	Caucasian	Serum	69.0/70.1	81/81	Case-control	RIA	Linco Research	7
Solomon et al.	2008	Pancreatic cancer	USA	Caucasian	Serum	58.0/58.0	311/510	Case-control	Elisa	Millipore	8
Krechler et al.	2011	Pancreatic cancer	Czech Republic	Caucasian	Plasma	51.9/64.5	64/64	Case-control	RIA	DRG Inc.	8
Grote et al.	2012	Pancreatic cancer	Germany	Caucasian	Serum	58.0/60.0	452/452	Nested case-control	Other	R&D Systems	8
Bao et al.	2013	Pancreatic cancer	USA	Caucasian	Plasma	NR	468/1080	Nested case-control	Elisa	ALPCO Diagnostics	8
Goktas et al.	2005	Prostate cancer	Turkey	Asian	Plasma	65.8/62.2	30/36	Case-control	RIA	Linco Research	8
Goktas et al.	2005	Prostate cancer	Turkey	Asian	Plasma	65.8/65.0	30/41	Case-control	RIA	Linco Research	8
Baillargeon et al.	2006	Prostate cancer	USA	Caucasian	Serum	63.5/63.2	125/125	Nested case-control	Other	Luminex	7
Michalakis et al.	2007	Prostate cancer	Greece	Caucasian	Serum	74.0/64.0	75/150	Case-control	RIA	Linco Research	5
Michalakis et al.	2007	Prostate cancer	Greece	Caucasian	Serum	74.0/70.0	75/75	Case-control	RIA	Linco Research	5
Housa et al.	2008	Prostate cancer	Czech Republic	Caucasian	Serum	63.6/70.5	43/25	Case-control	RIA	Linco Research	5
Grosman et al.	2010	Prostate cancer	Argentina	Caucasian	Serum	NR	25/25	Case-control	RIA	Linco Research	7
Li et al.	2010	Prostate cancer	USA	Caucasian	Plasma	59.0/58.6	620/599	Nested case-control	RIA	Linco Research	7
Dhillon et al.	2011	Prostate cancer	USA	Caucasian	Plasma	57.9/57.5	1286/1267	Nested case-control	RIA	Linco Research	8
Lopez Fontana et al.	2011	Prostate cancer	Argentina	Caucasian	Serum	63.8/64.9	35/35	Case-control	Elisa	Linco Research	6
Al Khaldi et al.	2011	Prostate cancer	Kuwait	Asian	Plasma	59.0/60.0	14/68	Case-control	Elisa	Linco Research	7
Touvier et al.	2013	Prostate cancer	France	Caucasian	Plasma	54.9/51.5	156/1024	Nested case-control	Elisa	R&D Systems	9
Tewari et al.	2013	Prostate cancer	India	Asian	Blood	66.5/65.7	95/95	Case-control	Other	NR	5
Spyridopoulos et al.	2012	Renal cancer	Greece	Caucasian	Serum	61.5/60.7	60/236	Case-control	RIA	Beth Israsel Deaconess Medical Center	8
Liao et al.	2013	Renal cancer	Finland/USA	Caucasian	Serum	57/57	273/273	Nested case-control	Elisa	Millipore	9
Liao et al.	2013	Renal cancer	Canada/USA	Caucasian	Serum	NR	768/917	Case-control	Elisa	Millipore	9
Mitsiades et al.	2011	Thyroid cancer	USA	Caucasian	Serum	51.2/55.4	175/107	Case-control	RIA	Beth Israsel Deaconess Medical Center	5
Guo et al.	2013	Tongue cancer	China	Asian	Serum	57.2/52.7	59/50	Case-control	Elisa	Adipobiotech	8

Abbreviations: NR, not reported; Elisa, enzyme-linked immunosorbent assay; RIA, radioimmunoassay.

### Circulating adiponectin levels and carcinogenesis

**Figure 2:**
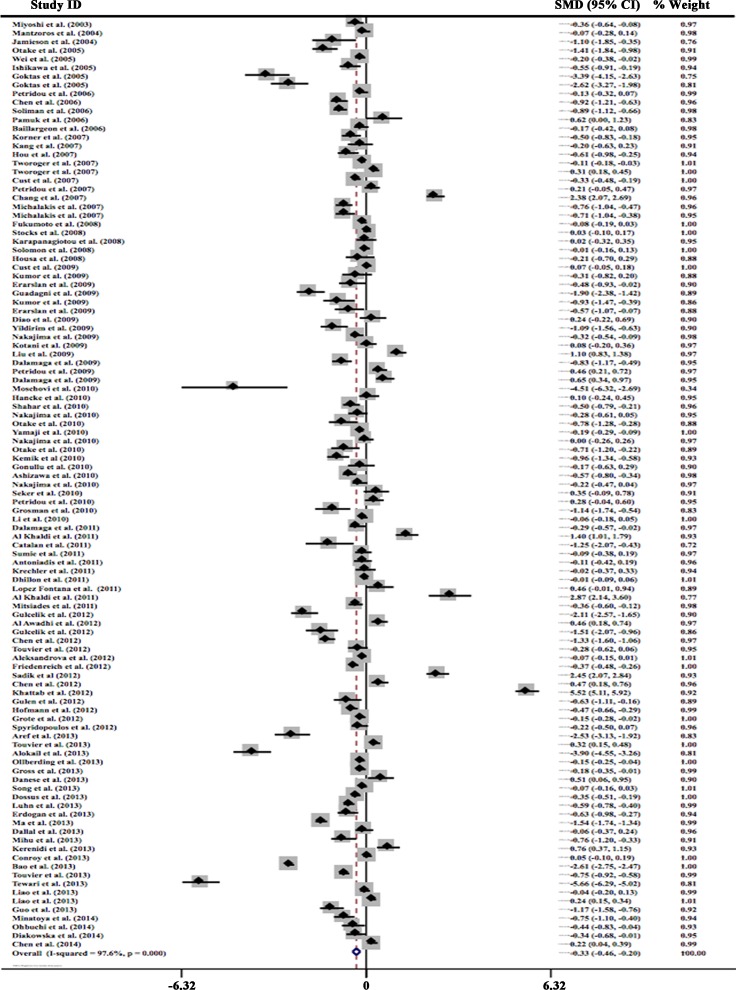
Forest plot of studies in circulating total adiponectin and cancer risk The combined SMD and 95% CIs were calculated through a random-effect model.

Data from 107 studies were analyzed in a random-effect model to compare circulating adiponectin levels in people with different cancers and controls. Results showed that circulating adiponectin levels in cancer cases were significantly lower than in the controls with a pooled SMD of −0.334 μg/ml (95% CI, −0.465 to −0.203, *P* = 0.000). Statistically significant amount of heterogeneity was observed across these studies (I^2^ = 97.6%, *P* < 0.0001), so subgroup analysis was carried out next. These results were presented in Figure 2.

**Figure 3:**
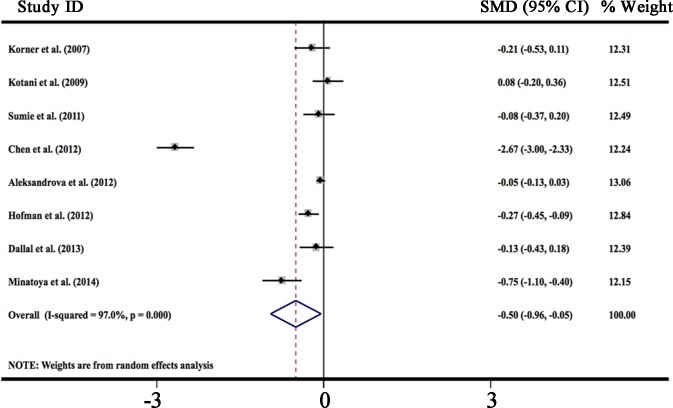
Forest plot of studies in circulating high molecular weight adiponectin and cancer risk The combined SMD and 95% CIs were calculated through a random-effect model.

HMW-adiponectin is the dominant form of adiponectin in plasma and correlates with cardiovascular disease, insulin resistance, and obesity [7, 113, 114]. But few studies have evaluated the relationship between circulating HMW-adiponectin levels and cancer risk. We analyzed data from 8 studies in a random-effect model to compare circulating HMW-adiponectin levels in people with different cancers [33, 56, 58, 72, 83, 94, 107, 108]. Results showed that circulating HMW-adiponectin levels in cancer cases were significantly lower than in the controls with a pooled SMD of −0.502 μg/ml (95% CI, −0.957 to −0.047, *P* = 0.000), which is consistent with the results derived from total adiponectin levels. Statistically significant amount of heterogeneity was observed across these studies (I^2^ = 97.0%, *P* < 0.0001). These results were presented in Figure 3.

### Subgroup analysis and meta-regression

Stratified subgroup analysis was performed to evaluate the potential sources of heterogeneity including ethnicity, cancer type, study design, blood sample, assay method, study size, study quality and mean age of cancer patients (Table 2). Lower levels of circulating adiponectin were observed in both Asian (SMD −0.555, 95% CI, −0.812 to −0.298) and Caucasian people (SMD −0.269, 95% CI, −0.400 to −0.138). Similar results were also presented in people with breast (SMD −0.334, 95% CI, −0.543 to −0.126), colorectal (SMD −0.496, 95% CI, −0.653 to −0.339), endometrial (SMD −0.594, 95% CI, −0.825 to −0.363), prostate (SMD −0.892, 95% CI, −1.345 to −0.438), thyroid (SMD −0.358, 95% CI, −0.601 to −0.116), tongue (SMD −1.172, 95% CI, −1.580 to −0.764), gastroesophageal (SMD −0.278, 95% CI, −0.553 to −0.004) cancer, multiple myeloma (SMD −0.621, 95% CI, −0.966 to −0.276), and acute leukemia (SMD −0.594, 95% CI, −0.825 to −0.363). Notably, circulating adiponectin levels were higher in the patients with hepatocellular cancer than in controls among 7 studies included (SMD 1.385, 95% CI, 0.240 to 2.530).

**Table 2:** Subgroup analysis of the relationships between circulating adiponectin levels and study characteristics

Characteristics	Number of studies	Number (Case/control)	SMD	95% CI	Heterogeneity (I2)
**Ethnicity**					
Caucasian	58	14178/19758	−0.269	−0.400 to −0.138	96.8%
Asian	46	4845/5456	−0.555	−0.812 to −0.298	97.1%
African	3	296/461	1.821	−2.201 to 5.843	99.6%
**Cancer Types**					
Acute leukemia	3	290/230	−2.236	−4.418 to −0.054	97.3%
Multiple myeloma	2	247/421	−0.621	−0.966 to −0.276	69.7%
Breast cancer	20	4545/5292	−0.334	−0.543 to −0.126	95.5%
Colorectal cancers	23	4749/5591	−0.496	−0.653 to −0.339	91.3%
Endometrial cancer	11	1876/3281	−0.594	−0.825 to −0.363	92.8%
Prostate cancer	13	2609/3565	−0.892	−1.345 to −0.438	97.9%
Thyroid cancer	1	175/107	−0.358	−0.601 to −0.116	NA
Tongue cancer	1	59/50	−1.172	−1.580 to −0.764	NA
Hepatocellular cancer	7	742/1526	1.385	0.240 to 2.530	99.2%
Gastroesophageal cancer	7	578/491	−0.278	−0.553 to −0.004	78.1%
Hodgkin lymphoma	1	75/75	0.28	−0.041 to 0.602	NA
Non-Hodgkin lymphoma	3	421/679	0.316	−0.048 to 0.68	79.7%
Lung cancer	5	349/299	−0.085	−0.58 to 0.409	87.1%
Melanoma	1	55/165	−0.112	−0.418 to 0.193	NA
Pancreatic cancer	6	1448/2477	0.037	−1.207 to 1.281	99.6%
Renal cancer	3	1101/1426	0.021	−0.246 to 0.288	86.6%
**Study Design**					
Case-control study	86	11965/14210	−0.346	−0.505 to −0.188	97.2%
Nested case-control study	21	7354/11465	−0.290	−0.553 to −0.026	98.5%
**Blood samples**					
Serum	65	9171/11101	−0.335	−0.483 to −0.186	95.8%
Plasma	37	8303/12010	−0.238	−0.497 to 0.022	98.6%
NR	5	1845/2564	−1.072	−1.775 to −0.369	98.8%
**Assay methods**					
RIA	29	6190/7587	−0.316	−0.459 to −0.172	93.0%
Elisa	71	12179/16968	−0.266	−0.426 to −0.106	97.4%
Others	7	950/1120	−1.305	−3.113 to 0.502	99.5%
**Study size**					
≥100 patients	48	16057/21437	−0.135	−0.299 to-0.030	98.3%
<100 patients	59	3262/4238	−0.549	−0.825 to −0.273	96.5%
**Study quality**					
≥6	96	16352/22425	−0.334	−0.465 to −0.203	97.3%
<6	11	2967/3250	−0.267	−0.700 to 0.165	98.2%
**Patients’ age (mean)**					
≥60	44*	4770/6414*	−0.489	−0.689 to −0.288	95.6%
<60	47*	9782/12935*	−0.194	−0.383 to −0.004	97.7%

Abbreviations: NA, not assessable.

* There are 91 studies with 14,552 cases and 19,349 controls reported the mean age of cancer patients.

Additionally, adiponectin was significantly lower in patients who used serum as test samples (SMD −0.335, 95% CI, −0.483 to −0.186), and in 37 studies who used plasma as testing samples, 26 studies showed the inverse relation of adiponectin to cancer risk. Assay method (radioimmunoassay or enzyme-linked immunosorbent assay) did not affect the results that circulating adiponectin was lower in cancer patients with pooled SMD of −0.316 and −0.266. Study size (more or less than 100 patients) did not change the result of estimated SMD either (SMD −0.135, 95% CI, −0.299 to −0.030; SMD −0.549, 95% CI, −0.825 to −0.273, respectively). Besides, no matter the mean age of cancer patients is older or younger than 60 years, decreased adiponectin levels were still exist in cancer patients (SMD −0.489, 95% CI, −0.689 to −0.288; SMD −0.194, 95% CI, −0.383 to −0.004, respectively).

Next we performed meta-regression to evaluate the effect of the above factors on the estimate of SMD. In meta-regression, none of the examined factors, such as ethnicity, cancer type, study design, blood sample, assay method, study size, study quality and mean age of cancer patients was proved to be significant contributing factors.

### Sensitivity analysis

Sensitivity analysis was performed by excluding one study at a time and calculating the pooled SMDs for the remaining studies. It was found that the combined SMDs were similar to one another and statistically significant. None of the studies influence the pooled results substantially in this analysis (Table 3).

**Table 3:** The pooled SMDs and 95% CIs of the included studies through sensitivity analysis

Study omitted	Estimate	95% CI
Miyoshi et al. (2003)	−0.33362126	−.46549249 to −.20175007
Jamieson et al. (2004)	−0.32790136	−.45929646 to −.19650623
Mantzoros et al. (2004)	−0.3366529	−.46877834 to −.20452745
Goktas et al. (2005)	−0.31018904	−.44067159 to −.1797065
Goktas et al. (2005)	−0.31467217	−.4453963 to −.18394804
Wei et al. (2005)	−0.33543056	−.46779135 to −.20306975
Otake et al. (2005)	−0.32370359	−.45491788 to −.19248928
Ishikawa et al. (2005)	−0.33179379	−.4634814 to −.20010617
Petridou et al. (2006)	−0.33611172	−.4683494 to −.20387407
Baillargeon et al. (2006)	−0.33559188	−.46757615 to −.2036076
Chen et al. (2006)	−0.32800215	−.45950019 to −.1965041
Pamuk et al. (2006)	−0.34167045	−.47312284 to −.21021806
Soliman et al. (2006)	−0.32821506	−.45972851 to −.19670163
Cust et al. (2007)	−0.33429027	−.46701819 to −.20156233
Korner et al. (2007)	−0.33221245	−.4639549 to −.20046999
Kang et al. (2007)	−0.33505732	−.46672907 to −.20338558
Tworoger et al. (2007)	−0.33802846	−.47386777 to −.20218913
Tworoger et al. (2007)	−0.3405844	−.47277904 to −.20838977
Chang et al. (2007)	−0.35825887	−.48626736 to −.2302504
Michalakis et al. (2007)	−0.32968622	−.46133375 to −.19803868
Michalakis et al. (2007)	−0.33015183	−.46179458 to −.19850905
Hou et al. (2007)	−0.33114624	−.46280947 to −.19948301
Petridou et al. (2007)	−0.33918613	−.47100937 to −.20736288
Fukumoto et al. (2008)	−0.33727011	−.47093907 to −.20360115
Solomon et al. (2008)	−0.3375347	−.47031215 to −.20475723
Housa et al. (2008)	−0.33494586	−.46656513 to −.2033266
Karapanagiotou et al. (2008)	−0.337192	−.46895465 to −.20542936
Stocks et al. (2008)	−0.33803195	−.47080877 to −.20525511
Dalamaga et al. (2009)	−0.34321341	−.47464448 to −.21178232
Dalamaga et al. (2009)	−0.32902971	−.46060005 to −.19745934
Petridou et al. (2009)	−0.34161058	−.47322133 to −.2099998
Kotani et al. (2009)	−0.33791459	−.46976718 to −.20606196
Guadagni et al. (2009)	−0.31941918	−.45032975 to −.18850861
Kumor et al. (2009)	−0.32856214	−.46004361 to −.1970806
Erarslan et al. (2009)	−0.33168712	−.46326634 to −.20010787
Kumor et al. (2009)	−0.3339898	−.4655939 to −.2023856
Erarslan et al. (2009)	−0.33251002	−.46413583 to −.20088424
Cust et al. (2009)	−0.33854863	−.47171903 to −.20537826
Nakajima et al. (2009)	−0.33416247	−.4662253 to −.20209965
Diao et al. (2009)	−0.33897933	−.47058302 to −.2073756
Yildirim et al. (2009)	−0.3268407	−.45826575 to −.19541568
Li et al. (2009)	−0.34737712	−.47806501 to −.21668923
Moschovi et al. (2010)	−0.31926209	−.45011383 to −.18841037
Hancke et al. (2010)	−0.33801222	−.46974581 to −.20627865
Seker et al. (2010)	−0.34004903	−.47163537 to −.2084627
Nakajima et al. (2010)	−0.33507797	−.46703228 to −.20312366
Petridou et al. (2010)	−0.33972663	−.47141987 to −.20803338
Grosman et al. (2010)	−0.32690996	−.45831883 to −.19550107
Li et al. (2010)	−0.3373847	−.47090244 to −.20386696
Nakajima et al. (2010)	−0.3372006	−.46912611 to −.2052751
Otake et al. (2010)	−0.3304036	−.46196187 to −.19884537
Kemik et al (2010)	−0.3278496	−.45933568 to −.19636351
Gonullu et al. (2010)	−0.3353405	−.46698609 to −.20369488
Nakajima et al. (2010)	−0.33436027	−.46614832 to −.20257224
Ashizawa et al. (2010)	−0.33154425	−.4634259 to −.19966258
Shahar et al. (2010)	−0.3222657	−.4640207 to −.20043245
Otake et al. (2010)	−0.3297922	−.46132797 to −.19825645
Yamaji et al. (2010)	−0.33636427	−.47040036 to −.20232819
Antoniadis et al. (2011)	−0.3360277	−.46786067 to −.2041948
Dhillon et al. (2011)	−0.33877328	−.47420669 to −.20333987
Al Khaldi et al. (2011)	−0.3581396	−.48865175 to −.22762746
Sumie et al. (2011)	−0.33623996	−.46812296 to −.20435697
Catalan et al. (2011)	−0.3270525	−.4584052 to −.19569978
Dalamaga et al. (2011)	−0.33432293	−.46621501 to −.20243084
Al Khaldi et al. (2011)	−0.34966454	−.48045498 to −.21887414
Krechler et al. (2011)	−0.33685401	−.46860847 to −.20509957
Mitsiades et al. (2011)	−0.33370164	−.46567562 to −.20172767
Lopez Fontana et al. (2011)	−0.3409504	−.47248313 to −.20941767
Spyridopoulos et al. (2012)	−0.33505121	−.46693206 to −.20317033
Hofmann et al. (2012)	−0.33265379	−.46483427 to −.2004733
Gulen et al. (2012)	−0.33108562	−.46266881 to −.1995024
Sadik et al (2012)	−0.35871589	−.48782459 to −.2296071
Chen et al. (2012)	−0.32368076	−.45458078 to −.1927807
Gulcelik et al. (2012)	−0.32346013	−.45471603 to −.19220424
Aleksandrova et al. (2012)	−0.33816311	−.47353563 to −.20279059
Friedenreich et al. (2012)	−0.33428073	−.46782497 to −.20073651
Touvier et al. (2012)	−0.3405067	−.47251758 to −.20849583
Gulcelik et al. (2012)	−0.31726849	−.44793424 to −.18660273
Al Awadhi et al. (2012)	−0.34153	−.47313255 to −.2099274
Grote et al. (2012)	0.33630246	−.46936187 to −.20324306
Chen et al. (2012)	−0.34161338	−.47320184 to −.2100249
Khattab et al. (2012)	−0.38207525	−.50227177 to −.2618787
Dossus et al. (2013)	−0.33401754	−.46652448 to −.20151059
Bao et al. (2013)	−0.30136451	−.41626969 to −.18645933
Guo et al. (2013)	−0.3258861	−.45724642 to −.1945257
Touvier et al. (2013)	−0.33433828	−.46610662 to −.2025699
Liao et al. (2013)	−0.33713764	−.46957946 to −.20469585
Liao et al. (2013)	−0.3403554	−.47350773 to −.20720309
Conroy et al. (2013)	−0.33808553	−.47072488 to −.2054462
Touvier et al. (2013)	−0.3296572	−.46145904 to −.19785538
Kerenidi et al. (2013)	−0.3439351	−.4753255 to −.21254471
Danese et al. (2013)	−0.34142008	−.47294763 to −.20989256
Song et al. (2013)	−0.33765575	−.47181916 to −.20349233
Luhn et al. (2013)	−0.33139816	−.46341154 to −.19938481
Ma et al. (2013)	−0.32076678	−.45034227 to −.1911912
Dallal et al. (2013)	−0.33651593	−.4683443 to −.20468754
Alokail et al. (2013)	−0.30377382	−.43343174 to −.17411587
Ollberding et al. (2013)	−0.33671638	−.47059336 to −.20283943
Gross et al. (2013)	−0.33571425	−.46818775 to −.20324075
Aref et al. (2013)	−0.31502652	−.4457356 to −.18431742
Tewari et al. (2013)	−0.28841972	−.41580069 to −.16103874
Erdogan et al. (2013)	−0.33102214	−.46268752 to −.19935676
Mihu et al. (2013)	−0.3298324	−.46139839 to −.1982664
Chen et al. (2014)	−0.33950841	−.47162384 to −.20739301
Ohbuchi et al. (2014)	−0.33285877	−.46454135 to −.20117618
Minatoya et al. (2014)	−0.32982665	−.46143582 to −.19821748
Diakowska et al. (2014)	−0.33376125	−.46553349 to −.20198898
Combined	−0.33375105	−.46467104 to −.20283107

### Publication bias

**Figure 4:**
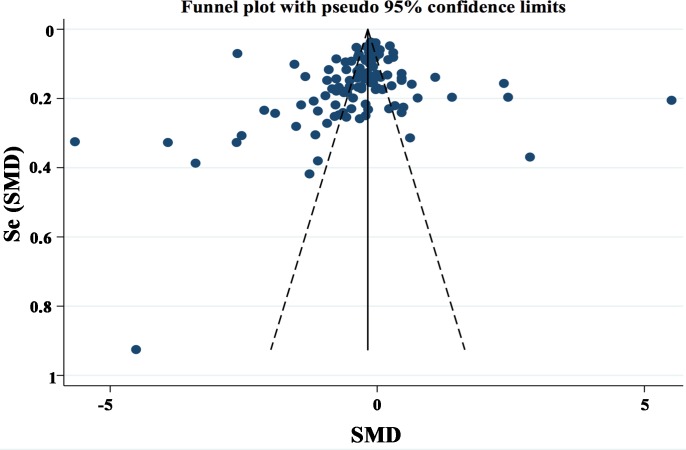
Funnel plot of lower adiponectin expression and cancer risk Circles indicate included studies.

**Figure 5:**
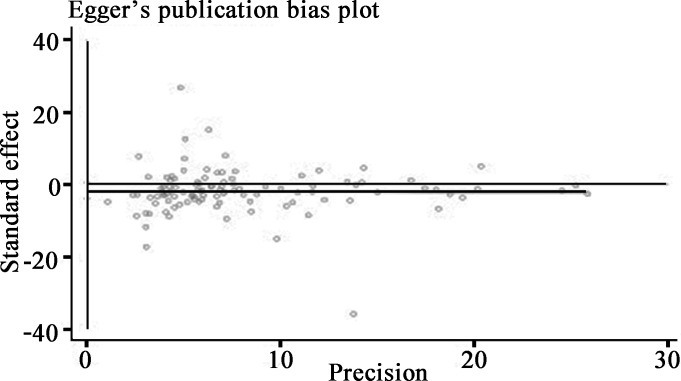
Egger's linear regression test for publication bias detection

Publication bias was assessed by funnel plot and Egger's regression test. Funnel plot shapes demonstrated a marginally asymmetrical distribution (Figure 4), accordingly we performed further analysis with Egger's test. The tested result (Figure 5) showed no evidence of publication bias (*P* = 0.123).

## DISCUSSION

By integrating 107 studies, our meta-analysis revealed that lower circulating adiponectin levels were associated with higher risk of cancers. Despite the existence of heterogeneity, the disparity of adiponectin levels between malignant individuals and controls reveals the potential ability of adiponectin to serve as a biomarker for early detection of cancers.

Aberrant adiponectin secretion is associated with tumor progression, metastasis and overall prognosis. Two previous meta-analysis indicated that lower adiponectin levels were associated with higher risk of breast cancer, colorectal cancer and colorectal adenoma [115, 116]. By synthesizing 107 studies involving 19,319 cases with different malignancies, the present meta-analysis estimate the inverse association between circulating adiponectin levels and cancer risk. Moreover, through subgroup analysis, we identified that this inverse relation of adiponectin to cancer risk might be more meaningful in breast, colon, endometrial, prostate, and gastroesophageal cancers. Besides, adiponectin levels tend to decrease as tumor stage increases in gastric cancer [62]. Kang et al. also indicate that breast cancer patients with less than the median adiponectin levels are easy to develop lymph node metastasis [82]. Low adiponectin level is the independent predictor of unfavorable prognosis in colorectal cancer [117]. These findings demonstrate that adiponectin is not only associated with cancer risk, but also correlated with tumor progression. Additionally, in our included 107 studies, 8 studies evaluated the relationship between circulating levels of adiponectin subtypes and cancer risk. The changing trend of total adiponectin was almost same with the three adiponectin subtypes in cancer patients, especially with HMW-adiponectin, that it is inversely associated with cancer risk.

Circulating adiponectin levels are affected by various factors, including inflammatory, dietary, hormonal, genetic, and medicine. One of possible explanations for decreased adiponectin levels in malignancies is the sustained inflammatory status of cancer patients leads to the increased proinflammatory cytokines such as TNF-α and IL-6, which are all reported to suppress adiponectin transcription and translation in adipocyte cell line [118, 119]. Besides, in obesity-related cancers, adiponectin may control its own production through a negative feedback loop during the development of obesity [120]. Moreover, dietary with lower intake of fiber and magnesium can also reduce circulating adiponectin levels [121].

However, elevated adiponectin levels are also reported in hepatocellular carcinoma. Since adiponectin is mainly degraded in the liver and adiponectin levels are elevated in advanced disease including cirrhosis and virus-related cancer [61, 122]. One possible explanation for increased adiponectin level in hepatocellular carcinoma might be due to deteriorated hepatic metabolism resulted from repeated necroinflammation and regeneration. Besides, conflicting results also exist in clinical studies of pancreatic cancer that both higher and lower adiponectin levels are reported to be associated with cancer risk [45, 50]. After reviewing the pancreatic cancer studies with higher levels of adiponectin, we found that almost half of them were accompanied with jaundice [45]. Since cholestasis would lead to the chronic liver deterioration, it is possible that increased adiponectin levels might be due to the reduced degradation.

The peripheral functions of adiponectin are mainly mediated through AdipoR1 and AdipoR2. The expression levels of AdipoRs vary between malignant tissues and their peritumoral normal counterparts. The upregulation of AdipoR1 and AdipoR2 are reported in gastric carcinoma [123], whereas decreased in prostate cancer tissues compared with the nonmalignant tissues [36]. Increased expression of AdipoRs may be the response of reduced circulating as well as local adiponectin levels and reduced expression suggests that the sensitivity of AdipoRs to adiponectin is decreased in tumor tissues. Yabushita et al. indicate that poor expression of AdipoR1 is associated with tumor invasion and lymph node metastasis, as well as poor prognosis in endometrial cancer patients [124]. A study of non-small cell lung cancer also indicates that patients with higher expression of AdipoR1 have longer overall survival and AdipoR2 expression is inversely correlated with tumor size [125]. Those findings further illustrate the protective role of adiponectin as well as AdipoRs and shed light on exploiting them for cancer therapy. Recently, AdipoRs agonist called 355ADP is identified and might represent a new strategy to replace low adiponectin level in cancer [126].

Despite the inverse correlation between adiponectin and various cancers, the underlying mechanisms of adiponectin in potential cancer suppression are still need to elucidate. Adiponectin decreases low density lipoprotein (LDL) receptor expression in breast cancer cells through promoting autophagic flux and inhibits LDL-cholesterol-induced tumor cell proliferation [127]. Adiponectin induces the phosphorylation of p53, a tumor suppressor, which renders cell cycle arrest and apoptosis in cancer cell lines [128]. Adiponectin also inhibits leptin-induced metastasis by downregulating JAK/STAT3 pathway, displaying an inverse correlation with cancer development [129]. In contrast, adiponectin promotes the angiogenesis in human chondrosarcoma by increasing vascular endothelial growth factor-A expression [130]. It is also reported to exert anti-apoptotic effects on pancreatic cancer cells through activation of AMPK/Sirtuin-1 signaling pathway [131]. Taken together, adiponectin might play a complicated role in carcinogenesis and progression of cancers.

Our study has some limitations that need to be addressed when interpreting the results. The significant heterogeneity was observed among the studies thus the conclusion should be more conservative. Although stratified analysis was conducted, none of the factors including ethnicity, cancer type, study design, blood sample, assay method, study size, study quality, and mean age of cancer patients were confirmed to contributing factors. Some possible reasons may partially explain this heterogeneity. Adiponectin levels are changed along with the tumor development. The tumor type, size, histological grade, and lymph node metastasis are the possible contributors caused heterogeneity. It is difficult for us to acquire the detailed information from the included studies. Besides, the subjects were from different regions and the lifestyle combined with diet was varied, which might influence the level of adiponectin. Since adiponectin is mainly secreted from adipose tissue, variables such as age, hormone receptor expression, menopausal status and BMI could contribute to the secretion and those factors were not fully deliberated for the complexity of tumor environment.

## CONCLUSIONS

In summary, the present study shows significant difference in circulating adiponectin levels between patients with malignancies and controls. Low circulating adiponectin level is associated with increased cancer risk, which suggests that adiponectin may serve as a potential biomarker for early detection of cancers considering its abundance in blood. Thorough understanding the roles of adiponectin and its receptors in the progression of cancers is helpful to cancer screening and promote individualized treatment.

## MATERIALS AND METHODS

### Search strategy

Based on the standard guidelines, a systematic search of English literature from Cochrane library, Wiley online library, PubMed was conducted to retrieve eligible studies until August 8, 2015. Searching terms included Medical Subject Heading (Mesh) and free text words “adiponectin”, “ADPN”, “Acrp 30”, “AdipoQ”, “GBP 28” or “apM1” in combination with “neoplasm”, “cancer”, “carcinoma”, “malignancy” or “tumor”. Furthermore, we manually searched references of relevant studies to add potential research to this meta-analysis.

### Inclusion and exclusion criteria

Studies were included if they met the following criteria: (i) full text case-control studies published in peer-reviewed journals evaluating the relationship between circulating adiponectin concentration and carcinogenesis; (ii) all cases were diagnosed as cancer by pathological biopsy or other medical methods with blood sample obtained before any therapies and all the controls were people without any cancers. (iii) circulating adiponectin level and standard deviation (SD) of it were provided or there were enough information to estimate them. Reviews, letters or animal experiments were excluded and articles without key information to carry on further analysis were also beyond consideration. Meanwhile, if replicated patient cohort was published in different studies, only the most recent or complete one was chosen. Since all the studies included were acquired from literature, ethics committee approval was not needed.

### Data extraction

Based on the checklist of MOOSE (Meta-analysis Of Observational Studies in Epidemiology) [132], two reviewers (Tai W and Peng Y) extracted the following data independently from eligible studies: the last name of first author, year of publication, geographic region, ethnicity, tumor type, study design, sample type, adiponectin assay method, number of patients and controls, assay source, mean ± SD of adiponectin concentration. Disagreement was resolved by discussion until the two reviewers reached a consensus.

### Quality assessment of included studies

Two reviewers (Tai W and Peng Y) independently assessed the quality of each included study according to the Newcastle-Ottawa Quality Assessment Scale (NOS) [133] ranges from 0 to 9 stars. Studies with more than 6 stars were considered as high-quality studies. Any disagreement was resolved by discussion and reevaluation.

### Statistical analysis

We acquired the mean ± SD of circulating adiponectin levels from cases and controls through three ways. The most accurate method was extracted them from the original research directly. However, a few studies presented the results as median values or standard error. In that case, we regarded median value as mean value considering the large sample size and calculated the SD value by using standard error and population number. If necessary, we contacted the author for detailed information. Standard mean differences (SMDs) and the corresponding 95% confidence intervals (CIs) of circulating adiponectin were calculated for all the eligible studies. Cochran's Q-test was performed to test the heterogeneity of included studies and *P* < 0.05 was considered statistically significant. Higgins I-squared statistic was applied to offer evidence of heterogeneity with I^2^ > 50% suggesting significant heterogeneity. The pooled SMD and 95% CI was calculated using a fixed-effects model if the heterogeneity was not significant, otherwise a random-effect model was employed and subgroup analyses and meta-regression were adopted to detect the potential cause of heterogeneity.

Sensitivity analysis was executed to detect the robustness of the results. Publication bias was evaluated by use of funnel plot and Egger's linear regression test. The Stata 13.0 software (Stata Corporation, College Station, TX, USA) was used to perform all the statistical analysis. All *P* values were two-sided.
